# Alzheimer’s-Related Peptide Amyloid-β Plays a Conserved Role in Angiogenesis

**DOI:** 10.1371/journal.pone.0039598

**Published:** 2012-07-09

**Authors:** D. Joshua Cameron, Cooper Galvin, Tursun Alkam, Harpreet Sidhu, John Ellison, Salvadore Luna, Douglas W. Ethell

**Affiliations:** 1 Molecular Neurobiology, Western University of Health Sciences, Pomona, California, United States of America; 2 College of Optometry, Western University of Health Sciences, Pomona, California, United States of America; 3 Graduate College of Biomedical Sciences, Western University of Health Sciences, Pomona, California, United States of America; 4 College of Osteopathic Medicine of the Pacific, Western University of Health Sciences, Pomona, California, United States of America; Pomona College, United States of America

## Abstract

Alzheimer’s disease research has been at an impasse in recent years with lingering questions about the involvement of Amyloid-β (Aβ). Early versions of the amyloid hypothesis considered Aβ something of an undesirable byproduct of APP processing that wreaks havoc on the human neocortex, yet evolutionary conservation - over three hundred million years - indicates this peptide plays an important biological role in survival and reproductive fitness. Here we describe how Aβ regulates blood vessel branching in tissues as varied as human umbilical vein and zebrafish hindbrain. High physiological concentrations of Aβ monomer induced angiogenesis by a conserved mechanism that blocks γ-secretase processing of a Notch intermediate, NEXT, and reduces the expression of downstream Notch target genes. Our findings allude to an integration of signaling pathways that utilize γ-secretase activity, which may have significant implications for our understanding of Alzheimer’s pathogenesis vis-à-vis vascular changes that set the stage for ensuing neurodegeneration.

## Introduction

Alzheimer’s disease (AD) is the most common cause of dementia in the elderly with more than 5 million cases in the USA [1]. Progressive cognitive impairment correlates with the appearance of insoluble Aβ deposits that begin in the entorhinal cortex, spread to the basal forebrain, and eventually affect most areas of the neocortex. These waxy deposits, or plaques, have an avascular core that is often surrounded by reactive astrocytes [2] and activated microglia [3]. Plaques and other dense core deposits coalesce from soluble Aβ, a peptide that is constitutively produced by most tissues in the body, with elevated levels occurring in the brain several years before the onset of clinically distinguishable AD [4], [5]. Previous versions of the amyloid hypothesis for AD have implicated Aβ oligomers and/or dense core deposits as responsible for synaptic pruning and neuronal losses [5].

Aβ is produced from amyloid precursor protein (APP) by a two-step process. First, β-amyloid cleaving enzyme (BACE) sheds a fragment of the extracellular domain producing a membrane-bound C99 intermediate. Second, C99 is cleaved within its transmembrane domain by γ-secretase, releasing 39–43 amino acid Aβ peptides from the outer cell surface, as well as an Aβ intracellular domain (AICD) [6]. Clinical efforts to reduce Aβ production in AD patients have focused on inhibitors of BACE or the γ-secretase complex, but recent trials were halted when it was found that γ-secretase inhibitors (GSI) exacerbate AD progression [7].

Disappointing results from a growing list of clinical trials has led many to question the validity of the amyloid hypothesis and re-examine the significance of Aβ in AD pathogenesis [8]. While it is true that APP-deficient mice are viable and fertile, they have substantial motor and behavioral problems that make them unlikely to survive outside a laboratory setting. APP knockdown zebrafish embryos are stunted and have movement problems [9]. Indeed, evolutionary conservation of the Aβ1–42 sequence within APP suggests this peptide has served an important role in survival and reproductive fitness throughout vertebrate evolution and was optimized for terrestrial life ∼350 million years ago. We previously put forth an idea that several aspects of AD pathology connote a physiological role for Aβ in vascular remodeling [10].

In addition to APP, γ-secretase processes more than 20 other substrates that participate in a diverse array of biological functions [11] such as cell-to-cell adhesion [12], differentiation [13], [14], and angiogenesis [15]. Vascular stability is one of the more consequential processes that rely on γ-secretase activity, by way of Notch signaling [16]. Binding of Delta-like ligand to Notch facilitates its cleavage by ADAM10 to produce Notch extracellular domain truncated (NEXT), which is analogous to the C99 fragment from APP. NEXT is then processed by γ-secretase releasing an extracellular fragment, akin to Aβ, as well as Notch intracellular domain (NICD) – similar to AICD – that translocates to the nucleus and drives transcription of Notch target genes, including *Hes-1* and *Hey-1* [17]. Inhibitors of Notch signaling and/or γ-secretase activity are well-known to suppress the expression of Notch target genes and de-repress angiogenesis [18], [19].

Analysis of blood vessels that lie in-between the plaques of APP23 mice – the AD mouse model - revealed dense, highly branched blood vessel networks that contained dead-ends and circular loops [20], similar to the inefficient networks that form with in response to γ-secretase inhibitors (GSI). Blood vessel stability is reinforced by Notch signaling, with disruptions causing endothelial shaft cells to adopt tip cell morphology in a process that is regulated by VEGF and Notch [19]. Tip cells from venous and arterial sides meet and fuse to form new blood vessels.

We have investigated if changes in γ-secretase activity might provide a link between high concentrations of Aβ and aberrant vascular structures that occur together in AD pathology. We show that human Aβ induces blood vessel branching in human endothelial cells and zebrafish brain, establishing that the underlying mechanism has remained largely unchanged since amphibians diverged from bony fishes approximately 350 million years ago.

## Results

**Figure 1:**
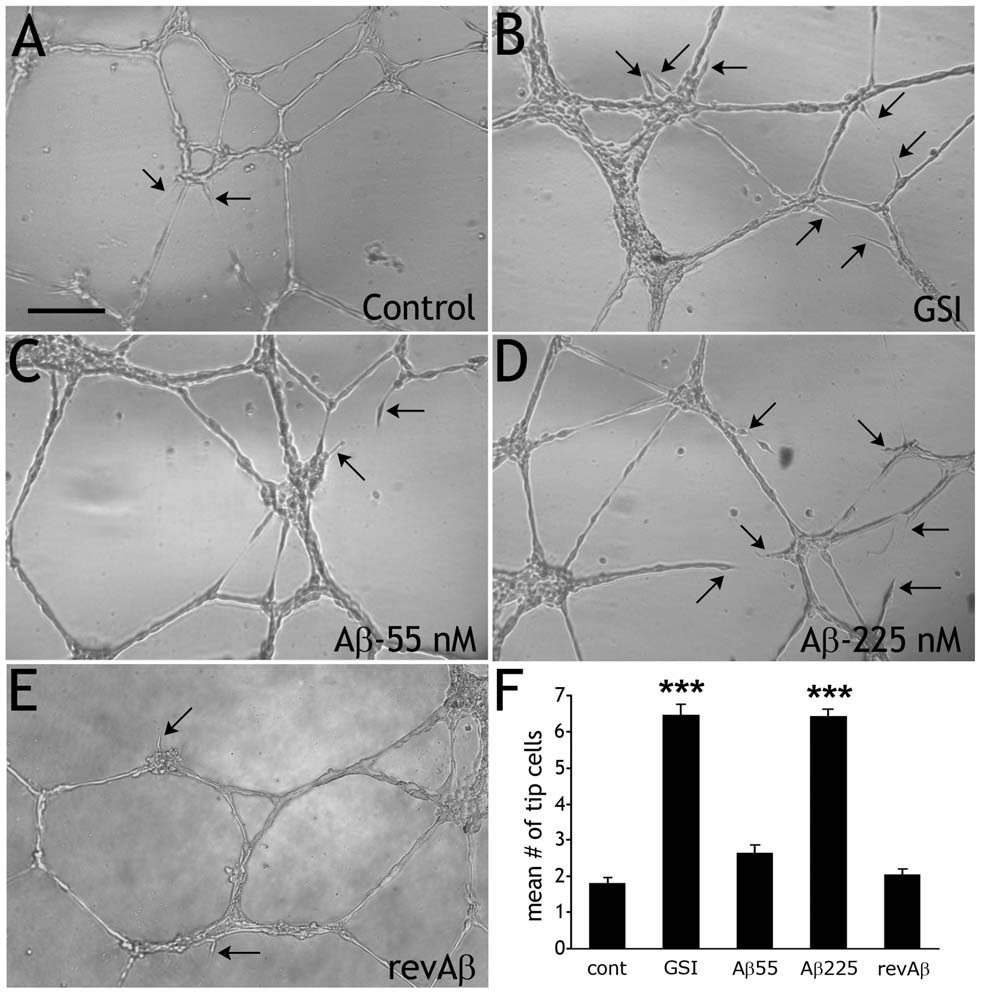
Aβ monomer induces tip cell formation in HUVEC. A) HUVEC plated on a 3-D matrix spontaneously formed vessel-like tube structures after 4 h. Tip cells are indicated with arrows. B) Tip cell frequency was significantly higher in cultures treated with GSI (p = 4.6e−29). C) Cultures treated with 55 nM Aβ did not have significantly more tips than controls, but cultures treated with 225 nM Aβ (D) had significantly more tip cells (p = 2.3e−29). E) Treatment with reverse Aβ42-1 did not increase tip cell frequency. F) Histogram of mean tip cells in all cultures (*** = p<0.001 vs. control, one-way ANOVA with Bonferroni correction). Scale bar  = 250 µm, for A–E.

To determine if Aβ monomers affect Notch signaling and endothelial tip cell formation, we assessed tube branching with human umbilical vein endothelial cells (HUVEC) [21]. HUVEC cultured in a 3-dimensional (3D) substrate spontaneously formed vessel-like tubes within 4 h and continued to develop tip cells up to 12 h later (Fig. 1A). Treatment with γ-secretase inhibitor (GSI) for 12 h induced a significant increase in the number of tip cells and branches (Fig. 1B, F). Treatment of HUVEC with a modest physiological concentration of Aβ1–42 (Aβ) monomer (55 nM) had little effect on tip cell number over control (Fig. 1A, C, F), but a higher (physiological) concentration (225 nM) significantly increased tip cell formation (Fig. 1D, F). Importantly, a high dose of reverse Aβ_42-1_ (revAβ) peptide did not significantly increase tip cell formation (Fig. 1E, F). No signs of apoptotic death were seen in HUVEC treated with Aβ (55 or 225 nM), as determined by nuclear morphology (DAPI) or sib-diploid peaks analysis (flow cytometry).

**Figure 2:**
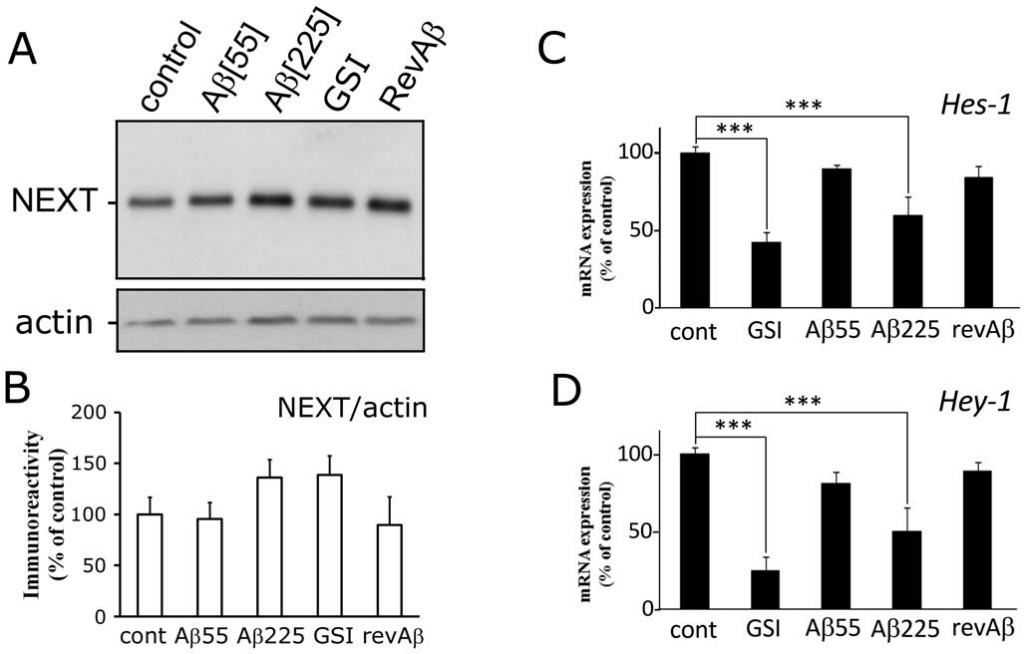
Aβ monomer causes HUVEC to accumulate NEXT and reduces mRNA levels of the downstream Notch targets *Hes-1* and *Hey-1*. A) Western blot of whole cell lysates from HUVEC cells treated with GSI (1 µM), Aβ (55 nM), Aβ (225 nM), reverse Aβ_42-1_ (225 nM), or PBS (control). Note the levels of NEXT (∼90 kD) are significantly higher in cells treated with GSI and both concentrations of Aβ. The highest level of NEXT was detected in HUVEC treated with 225 nM Aβ. B) Histograms showing mRNA transcript levels for *Hes-1* (B) and *Hey-1* (C) in treated HUVEC cells, as detected by qPCR. Messenger RNA levels for GSI-treated HUVEC were significantly lower than PBS-treated control cells. A high (225 nM) concentration of Aβ monomer reduced both *Hes-1* and *Hey-1* mRNA levels; whereas, moderate (55 nM) Aβ and revAβ (225 nM) did not (n = 9, triplicate wells in 3 separate experiments, *** = p<0.001 vs. control, one-way ANOVA with Bonferroni correction).

γ-secretase activity could be a common link between high levels of Aβ and dense, highly branched blood vessels, both of which occur in brain areas affected by AD pathology. Most enzymatic processes have some form of feedback inhibition to limit production, so we asked if high levels of Aβ might disrupt γ-secretase activity and reduce its processing of the Notch intermediate NEXT [10]. HUVEC displayed baseline Notch signaling, as indicated by the presence of NEXT protein (Fig. 2A) and the expression of Notch target genes, *Hes-1* and *Hey-1* (Fig. 2B). As expected, GSI blocked NEXT processing (Fig. 2A) and reduced mRNA levels of *Hes-1* and *Hey-1* (Fig. 2B, C). Human Aβ also increased NEXT in a dose-dependent manner in HUVEC (Fig. 2A). HUVEC treated with a high physiological concentration of Aβ also had significantly lower mRNA levels of *Hes-1* and *Hey-1* when compared with controls (Fig. 2B, C). These findings demonstrate that high levels of Aβ monomer can disrupt Notch signaling by reducing NEXT processing and suppress downstream Notch target genes.

**Figure 3:**
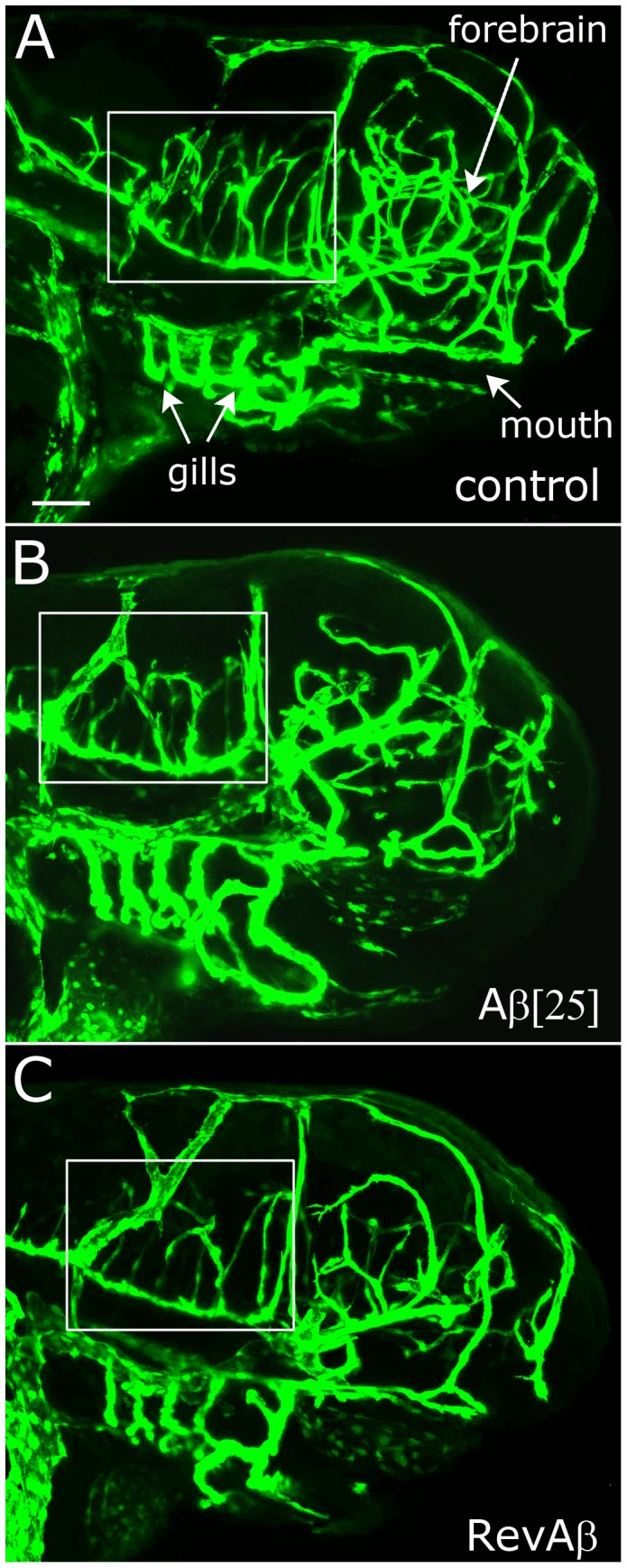
Morphology and vascular imaging of GFP-expressing zebrafish embryos. A) Projection image of a 3 dpf embryo using images captured by confocal microscopy. B, C) Comparable projection of a 3 dpf embryo treated with Aβ (25 µg/mL) and Rev Aβ, respectively. Boxes indicate the region analyzed for CtA branching, shown in detail in figure 4. Scale bar  = 100 µm, for A–C.

**Figure 4:**
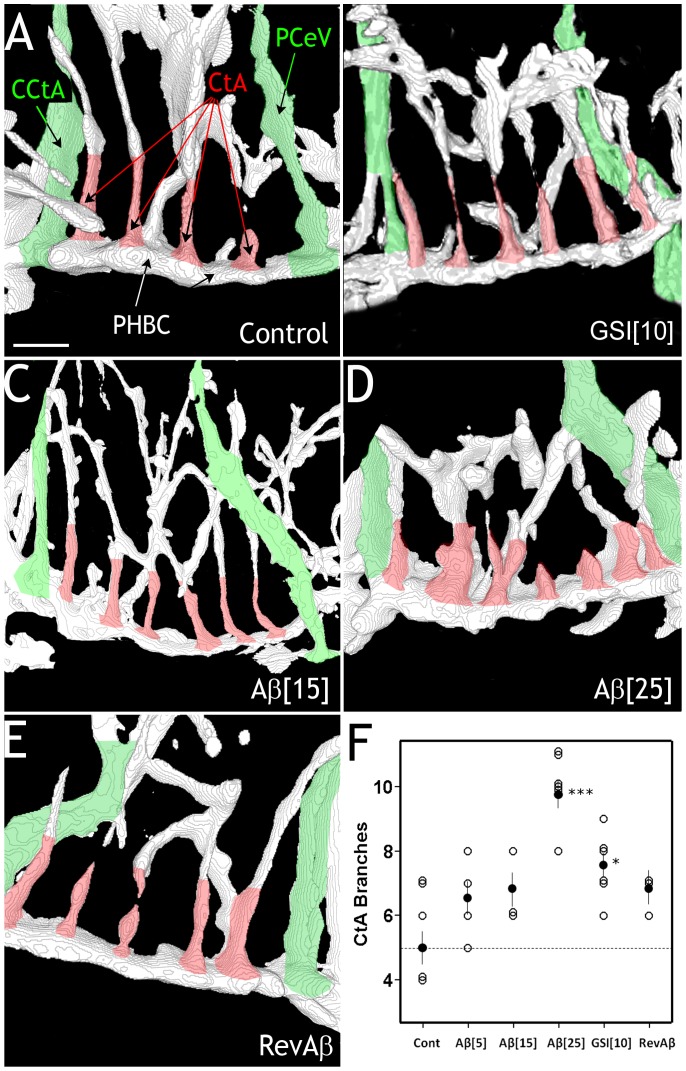
Analysis of CtA blood vessels in 3 dpf embryos. A) Digitized image of the regions highlighted in figure 3 in a control (untreated) 3 dpf embryo. B) CtA branching in GSI-treated (10 µg/mL) embryo at 3 dpf. C) CtA branching in an embryo treated with 15 µg/mL Aβ. D) CtA branching in an embryo treated with 25 µg/mL Aβ at 3 dpf. E) RevAβ did not significantly increase CtA branching. F) Scatter plot of CtA branches in all embryos in each condition (open circle). In the case of multiple values at given value for each condition, the circles were slightly offset. The mean for each group is shown as a filled circle and error bars represent standard errors of the mean. A dashed line indicates the mean value of the controls for comparison across the other treatments. Asterisks indicate significant differences with the untreated control group (*** = p<0.001; * = p<0.05 one-way ANOVA with Bonferroni correction). Scale bar  = 25 µm, for A–E. (CtA – cerebral artery, CCtA – common CtA, PCeV – posterior cerebral vein, PHBC - posterior hindbrain channel).

To explore the effects of Aβ monomer on blood vessel branching in vivo we used transgenic zebrafish that express green fluorescent protein (GFP) in vascular endothelial cells [22], which provide a format to image the vascular system of an intact embryo (Fig. S1). Confocal imaging of the cerebrovasculature was done on zebrafish embryos at 3 and 4 days post-fertilization (dpf) (Fig. 3A), and image stacks were rendered to produce 3-D maps of vascular structures in the head (Fig. 4A–E, Movie S1). Our analysis of 3-D maps from control embryos revealed a consistent pattern for central artery branches (CtA) entering the primordial hindbrain channel (PHBC) between two readily identifiable landmarks (Fig. 4A, E, F). At 3 dpf, control embryos had ∼5 CtA branches between the common CtA (CCtA) and the posterior cerebral vein (PCeV) (Fig. 4A, E).

Embryos treated with high levels of GSI had a characteristic curved morphology and smaller bodies (Fig. S2). Although embryos treated with 25 µg/mL GSI appeared to have increased vascular branching throughout the brain, their altered morphology made for variable scoring of CtA branches, so lower concentrations of GSI (5 and 10 µg/mL) were used that did not cause extreme morphological effects. Embryos treated with 10 µg/mL GSI had significantly more CtA branches than controls (Fig. 4B, F). Embryos treated with 5 or 15 µg/mL of monomeric Aβ had more CtA branches at 3 dpf than controls, with what appeared to be a dose-dependent effect, but the differences were not statistically significant (Fig. 4C, F). However, 3 dpf embryos that had been treated with a high concentration of Aβ (25 µg/mL) had significantly more CtA branches on the PHBC, and many of those branches bifurcated (Fig. 4D, F). RevAβ (42-1) (15 µg/mL) did not significantly increase CtA branching over control Aβ1–42 (Fig. 4E). These findings demonstrate that monomeric Aβ can induce blood vessel branching in the cerebrovasculature of developing zebrafish.

## Discussion

**Figure 5:**
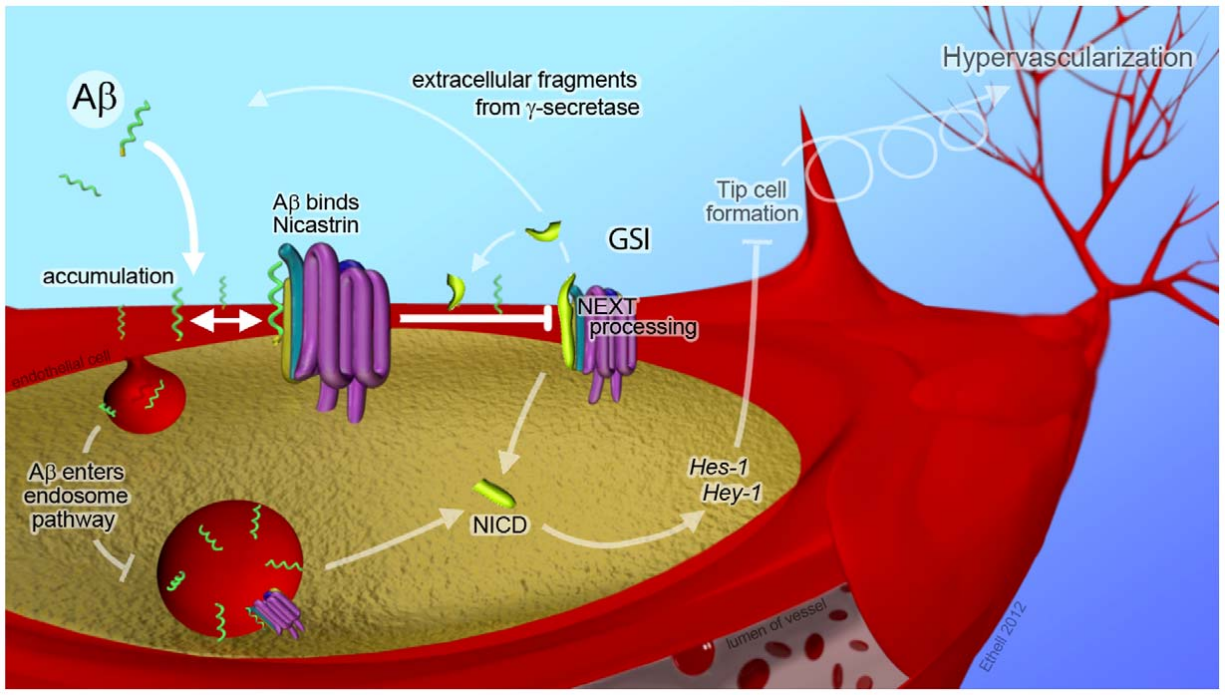
Proposed mechanism for hypervascularization in response to high concentrations of Aβ. High levels of Aβ (green helix) develop in the brain prior to the onset of Alzheimer’s pathology (top left). Longer forms of Aβ have more hydrophobic amino acid residues (gold) on remnant transmembrane domain, thereby increasing transient insertion (downward arrow) into the plasma membranes of nearby cells including endothelial cells (red). Elevated tissue levels of Aβ cause an accumulation of Aβ on the surface of the cell (several helices). Some peptide enters the endosome pathway (left). The amino termini of inserted Aβ peptides are identical to the Nicastrin-binding region of the APP intermediate C99 (not shown) and transiently bind to the γ-secretase complex (two sided arrow). Competition for Nicastrin binding and γ-secretase access reduces the processing of other substrates such as NEXT (shown). Interference with NEXT processing inhibits the production of NICD, which de-represses transcription factors such as Hes-1 and Hey-1. GSI has a similar effect. De-repression of NICD targets causes endothelial shaft cells to adopt tip cell morphology (upper right). Tip cells lead to formation of new blood vessels, and cycling of this process (looped arrow) over years and decades leads to hypervascularization (top right). This process may also occur with other extracellular cleavage (ex) products of γ-secretase (shown) creating feedback that tempers γ-secretase activity.

Our findings suggest a highly conserved mechanism (Fig. 5) in which Aβ monomers stimulate angiogenesis by a process that may be critical to our understanding of AD pathogenesis. Alzheimer’s risk is increased by conditions that hinder vascular flow, including atherosclerosis, diabetes, and a sedentary lifestyle [23]. Those factors reduce Aβ efflux from the brain and increase blood vessel branching, which over many years could result in the formation of dense, highly branched blood vessel networks, as occur in AD brain and in Aβ over-expressing mouse models [24], [25]. Early angiogenic sprouting could temporarily restore flow rates and reduce brain Aβ, but persistently high levels of longer Aβ species would eventually result in hyper-vascularization and a reduction of perfusion efficiency. Those changes could set the stage the accumulation of much higher levels of Aβ in the parenchyma that could cause coalesce into plaques and trigger neurodegeneration; further, it could diffuse into adjacent neural tissue where is would change the vascular structure and cause the cycle to repeat and spread AD pathology. It is worth noting that AD pathology spreads continguously from entorhinal cortex, hippocampus, basal forebrain and neocortex. This mechanism may explain the temporal nature of Alzheimer’s disease with initial symptoms that often stabilize for a few years before rapid deterioration.

A possible mechanism for feedback inhibition of Aβ peptides on the γ-secretase complex may rest with hydrophobic residues near the carboxyl terminus. The amino-terminus of C99 is bound by Nicastrin, which mediates substrate entry into the γ-secretase complex [26]. Once inside the complex, internal amino acids from both C-terminal and N-terminal fragments (CTF and NTF, respectively) of Presenilin mediate substrate cleavage. Initially, the substrate is cleaved at the cytoplasmic interface [16], releasing the intracellular fragment, and then amino acids are removed from the transmembrane region before the extracellular fragment is released by the complex. Variability of Aβ species (39–43 amino acids) depends on the strength of those interactions and may explain why AD-linked mutations in Presenilin-1 cause more Aβ1–42 to be produced [27], [28]. Hydrophobic residues from long Aβ species (Aβ1–42 and Aβ1–43) may transiently insert into the plasma membrane of cells, and possibly enter the endosome pathway (Fig. 5), resulting in slower tissue clearance rates than Aβ species with short hydrophobic tails (Aβ_1–39_ and Aβ_1–40_). Importantly, the amino-termini of these peptides project outward and contain the same Nicastrin-binding motif as C99. Brains that produce higher levels of Aβ could accumulate enough peptide on the outer surface, or within endosomes, to compete for Nicastrin with NEXT. This effect may also happen with the extracellular products of other γ-secretase substrates including, cadherin intermediates.

Amyloid-Notch cross-talk [21] may explain the failure of γ-secretase inhibitors in human trials for Alzheimer’s disease, and could clarify the source of vascular complications that have been reported with anti-Aβ antibody therapies [29]. Our findings suggest that high levels of Aβ monomer may set the stage for ensuing Alzheimer’s pathology by disrupting γ-secretase processing of NEXT and, as such, GSI compounds should exacerbate this effect. Indeed, clinical trials with the GSI Semagacestat worsened AD symptoms in some subjects, who did not improve when administration of the drug was stopped [30]. Antibodies directed against Aβ have been more promising in clinical trials, though there are concerns about the frequency of MRI signal intensity shifts, indicative of micro-hemorrhages that were first reported in mouse studies with similar antibodies [31]. If anti-Aβ antibodies are transported across brain endothelial cells they would quickly encounter Aβ in the perivascular-interstitial space. Antibody binding would thereby prevent Aβ from interacting with Nicastrin, resulting in a loss of γ-secretase inhibition on the blood vessels in AD affected brain areas. A consequent burst of γ-secretase activity acting on the backlog of unprocessed substrates, including NEXT and cadherin intermediates, would strongly induce NICD and TCF/LEF target genes. Such a combination of factors may disrupt VE−/E-cadherin-mediated interactions between endothelial cells, leading to transient leakiness of the blood-brain barrier, as was reported for Bapineuzumab in the early stages of treatment [32].

γ-secretase is a major integrator of cellular activity and the mechanism described here suggests that its extracellular cleavage products, such as Aβ, may feedback to regulate γ-secretase dependent signaling pathways. Future studies will determine how feedback inhibition from the spectrum of γ-secretase products may impact biological processes that rely on this important complex.

## Materials and Methods

### Reagents

Human Aβ1–42 was purchased from BioMer Technology (Pleasanton, CA). The product was received as a lyophilized powder that was re-suspended by first adding water, and titrating with 1N NaOH until the pH was neutralized and the suspension dissolved completely (i.e. the solution became clear). The solution was then mixed with 10× PBS, diluted to a final volume of 1 mg/ml (1× PBS final) and stored as frozen aliquots. This protocol produced solutions that consist primarily of primarily Aβ monomers, as established by a single peak with HPLC [33], although they can be reformed into neurotoxic oligomers when incubated at high-concentration for 3–5 days at 37°C [33], [34]; however, in the present study we used only freshly prepared solutions of Aβ monomer at concentrations and temperatures that were too low to cause significant oligomers formation. RevAβ (Aβ42-1) was obtained from Sigma-Aldrich and re-suspended the same way. γ-secretase inhibitor (GSI IX/DAPT {N-[N-(3,5-Difluorophenacetyl-L-alanyl)]-S-phenylglycine t-Butyl Ester} in solution), purchased from EMD Biosciences (San Diego, CA), diluted to 5 mg/mL with DMSO, and stored as aliquots at −20°C. All other reagents were from Sigma unless otherwise noted.

### Angiogenesis Assay

Human umbilical vein endothelial cells (HUVECs; Lonza) were cultured in EGM-2 complete medium (HUVEC; Lonza) in a humidified incubator at 37°C, 5% CO_2_. The cells were sub-cultured, fed every 2 days, and used for experiments between passages 4 and 6. Matrigel (BD Biosciences) was polymerized in a 96-well plate (50 µL/well) for 1 h at 37°C before being seeded with the HUVEC (4800 cells/cm^2^, or 1.5×10^4^/well) in 50 µL EGM-2 complete medium that included 50 nM β-phorbol 12-myristate 13-acetate (PMA). Cells were allowed 4 h for preliminary tube formation before receiving different treatments in the same culture medium (50 µL/well). Following a 12 h treatment period, tube formation was documented using an inverted microscope with a 4× objective and pictures were captured with a digital camera. Numbers of tips cells were counted in each of 25 images. Significance was calculated with ANOVA with Bonferroni post-hoc correction. Experiments were replicated 4 times with similar results.

### Western Blots

HUVEC cells were treated with PBS, GSI or Aβ monomers (55 nM or 225 nM) for 12 h before collection and whole cell lysis (100 mM Tris, pH = 7.4, 0.5 M NaCl, 10% Triton X-100, 2 mM EDTA, protease inhibitor cocktail). Lysates were pelleted at 14,000×g for 10 min at 4°C, the supernatants isolated and boiled in reducing sample buffer, and then run on 10% Mini-PROTEAN TGX pre-cast gels (Biorad). The proteins were transferred to HybondECL (Millipore) and blocked for 1 h at room temperature in 5% skim milk. Primary antibody incubations were done overnight at 4°C using appropriate antibodies diluted in TBS/0.1% Tween-20. Primary antibodies and dilutions were as follows: Rabbit α-Notch1 (C44H11) - 1∶1000 (Cell Signaling Technologies), and Goat α-actin (C11) - 1∶200 (Santa Cruz Biotech). Blots were washed 3×10 min with TBS/0.05% Tween-20 and incubated with HRP-conjugated secondary antibodies for an hour at room temperature in a TBS/0.1% tween-20 buffer solution; secondary antibodies used were Anti-Goat HRP - 1∶25,000 (Rockland), Anti-Rabbit HRP - 1∶5000, Anti-Mouse HRP - 1∶5000 (GE Healthcare). After secondary antibody incubations, blots were washed 3×10 min in TBS/0.05% Tween-20, and then developed with ECL Detection reagent (GE Healthcare). For actin re-probing, membrane blots were washed in stripping buffer (2% SDS, 100 mM β-mercaptoethanol, 50 mM Tris-HCl, pH 6.8) for 15 min at 56°C, rinsed repeatedly with water, blocked with 5% skim milk, and then re-probed.

### Quantitative Real-time qPCR for HUVEC mRNA

HUVECs were plated in matrigel-coated 96-well or 24-well plates, and then treated as above. Total RNA from HUVECs was prepared using Trizol (Life Technologies) according to the manufacturer’s instruction. cDNA was synthesized using SuperScript VILO cDNA Synthesis Kit (Life Technologies) as recommended by the manufacturer. To examine mRNA expression of *Hes-1* and *Hey-1*, the following primers were used: *Hes-1* forward primer: 5′-ACGTGCGAGGGCGTTAATAC; *Hes-1* reverse primer, 5′-ATTGATCTGGGTCATGCAGTT; *Hey-1* forward primer, 5′-AGAGTGCGGACGATGGAAACT; *Hey-1* reverse primer, 5′-CGTCGGCGCTTCTCAATTATTCCT. All samples were normalized to the expression of actin using the primers: *Actin* forward primer, 5′-GTGGAGTCTACTGGTGTCTTC; *Actin* reverse primer, 5′-GTGCAGGAGGAGGCATTGCTTACA. Each reaction mixture contained 1× Power SYBR Green PCR Master Mix (Life Technologies). All the reactions were run in triplicate. The PCR amplification protocol was as follows: initial DNA Polymerase activation at 95°C for 10 min, followed by 40 cycles with denaturation at 95°C for 15 s, and annealing + extension at 60°C for 1 min. Amplification was performed in a StepOne Real Time PCR System (96-well format) (Life Technologies) and analyzed using the comparative Ct (2^-ΔΔCt^) method, with *Hes-1* and *Hey-1* normalized to *Actin* within each sample.

### Zebrafish

Tg (kdr:EGPF)s843 transgenic zebrafish, expressing GFP in vascular endothelial cells, were obtained from the Zebrafish International Resource Center/ZIRC (Eugene, OR), and maintained under standard conditions at 28.5°C on a 10 h dark - 14 h light cycle [35]. Embryos were staged in hours post-fertilization (hpf) and days post-fertilization (dpf) based on morphological features. Embryos were raised in E3 buffer (5 mM NaCl, 0.17 mM KCl, 0.33 mM CaCl_2_, 0.33 mM MgSO_4_) at 28.5°C. All animal husbandry and experiments were approved and conducted in accordance with guidelines set forth by the Institutional Animal Care and Use Committee of Western University of Health Sciences.

### Embryo Treatments

Zebrafish were de-chorionated at 24 hpf just prior to treatment. Treatment solutions were diluted in E3 buffer containing 0.003% 1-phenyl-2-thio-Urea (PTU) to inhibit pigment development. Treatments consisted of 5, 15, or 25 µg/mL monomeric Aβ, 25 µg/mL reverse Aβ_42-1,_ 25 µg/mL GSI (in DMSO) and equivalent volumes of DMSO or E3 buffer for control. The embryos remained in the treatment conditions until 3 dpf when they were fixed in 4% paraformaldehyde/PBS overnight.

### Zebrafish Imaging

Eyes were removed from the fixed embryos by grazing the membrane with a tungsten needle until they became dislodged. Zebrafish embryos were laid on their sides and mounted on cover glass using a drop of warm 1% agarose.

Confocal imaging was done with a Nikon A1 3-color confocal microscope using a 20× objective, taking image stacks from the lateral surface to midline that included the head of each embryo. Images were Gaussian smoothed, thresholded, and rendered in 3D using the Fiji distribution of ImageJ-3D. 3D viewing and rotation of the composite images were used to view and score central arteries (CtA) that emerge from the Basilar artery and project to the PHBC primordial hindbrain channel, using the CCtA and posterior cerebral vein (PCeV) as rostral and caudal landmarks, respectively. Only branches connecting to the PHBC were scored.

### Statistical Analysis

One-way ANOVA with Bonferroni post-hoc analysis was used to analyze both HUVEC experiments and zebrafish blood vessel branching. Data presented in all histograms is mean and standard error of the mean.

## Supporting Information

Figure S1 **Comparison of cerebrovascular structures in control zebrafish embryos at 3–7 dpf.** Embryos expressed GFP in endothelial cells and images were captured with a confocal microscope.
(TIF)

Figure S2 **Gamma-secretase inhibitor effects on morphology.** A) Dorsal view of 4 dpf embryos: control – top, Aβ [25] treated – middle, and GSI treated – bottom. B, C) Lateral view of control and GSI- treated 4 dpf embryos, respectively.
(TIF)

Movie S1 **Quicktime movie showing the 3D vascular structure and the location of CtA vessels in 3 dpf zebrafish embryo.** The animation starts with a bright field image of a 3 dpf zebrafish embryo for orientation purposes. Dissolving of this overlay reveals a 3D reconstruction of confocal images taken from an embryo that expressed GFP in all blood vessels. White vessels in the 3D image were green with GFP. The 3D animation stops on the vessels of interest in the brainstem. An overlaid digital map illustrates how CtA branches were identified and scored.
(MOV)
