# Supplementary figures and images for: Alzheimer’s-Related Peptide Amyloid-β Plays a Conserved Role in Angiogenesis
Source: PLoS One. 2012 Jul 9;7(7):e39598. doi: 10.1371/journal.pone.0039598 (PMC3392248; doi:10.1371/journal.pone.0039598)

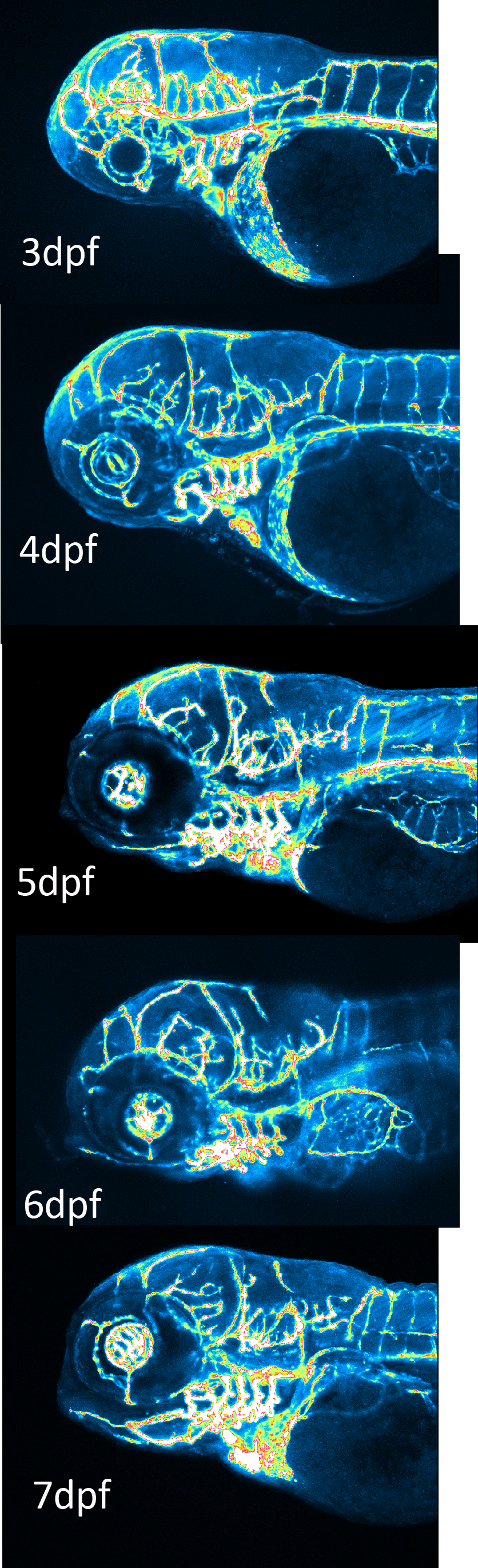

Supplement: Figure S1 — Comparison of cerebrovascular structures in control zebrafish embryos at 3–7 dpf. Embryos expressed GFP in endothelial cells and images were captured with a confocal microscope. (TIF) [file pone.0039598.s001.tif]

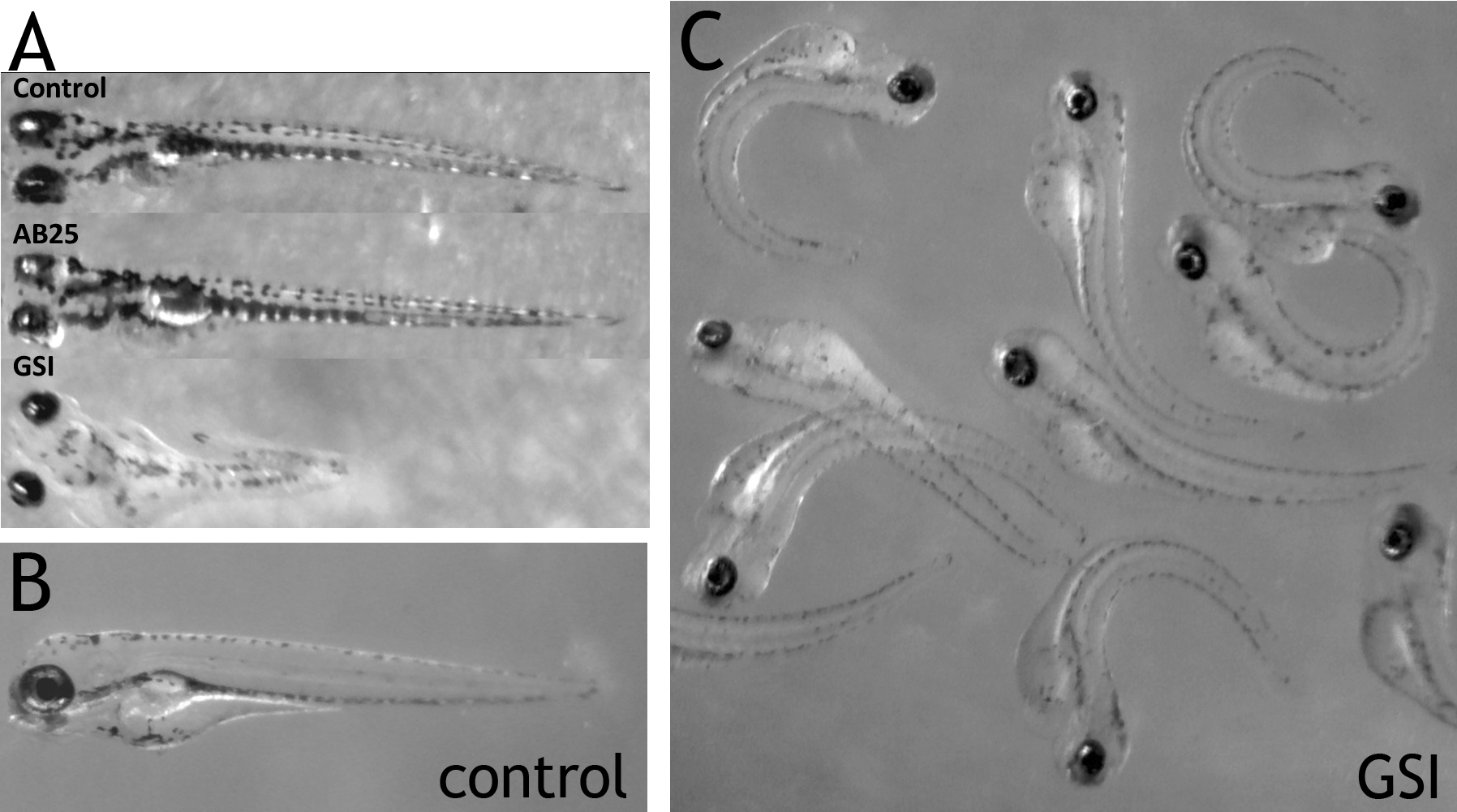

Supplement: Figure S2 — Gamma-secretase inhibitor effects on morphology. A) Dorsal view of 4 dpf embryos: control – top, Aβ [25] treated – middle, and GSI treated – bottom. B, C) Lateral view of control and GSI- treated 4 dpf embryos, respectively. (TIF) [file pone.0039598.s002.tif]
